# High-Titer Lactic Acid Production by *Pediococcus acidilactici* PA204 from Corn Stover through Fed-Batch Simultaneous Saccharification and Fermentation

**DOI:** 10.3390/microorganisms8101491

**Published:** 2020-09-28

**Authors:** Zhenting Zhang, Yanan Li, Jianguo Zhang, Nan Peng, Yunxiang Liang, Shumiao Zhao

**Affiliations:** 1 State Key Laboratory of Agricultural Microbiology, College of Life Science and Technology, Huazhong Agricultural University, Wuhan 430070, China; zhangzhenting@webmail.hzau.edu.cn (Z.Z.); lana463936202@163.com (Y.L.); zhangjianguo@hfut.edu.cn (J.Z.); nanp@mail.hzau.edu.cn (N.P.); fa-lyx@163.com (Y.L.); 2 School of Food and Biological Engineering, Hefei University of Technology, Hefei 230009, China

**Keywords:** lactic acid fermentation, *Pediococcus acidilactici* PA204, simultaneous saccharification and fermentation, corn stover

## Abstract

Lignocellulose comprised of cellulose and hemicellulose is one of the most abundant renewable feedstocks. Lactic acid bacteria have the ability to ferment sugar derived from lignocellulose. In this study, *Pediococcus acidilactici* PA204 is a lactic acid bacterium with a high tolerance of temperature and high-efficiency utilization of xylose. We developed a fed-batch simultaneous saccharification and fermentation (SSF) process at 37 °C (pH 6.0) using the 30 FPU (filter paper units)/g cellulase and 20 g/L corn steep powder in a 5 L bioreactor to produce lactic acid (LA). The titer, yield, and productivity of LA produced from 12% (*w*/*w*) NaOH-pretreated and washed stover were 92.01 g/L, 0.77 g/g stover, and 1.28 g/L/h, respectively, and those from 15% NaOH-pretreated and washed stover were 104.11 g/L, 0.69 g/g stover, and 1.24 g/L/h, respectively. This study develops a feasible fed-batch SSF process for LA production from corn stover and provides a promising candidate strain for high-titer and -yield lignocellulose-derived LA production.

## 1. Introduction

Lignocellulose, the most abundant global source of renewable biomass, is one of the most important raw materials for biofuel and biochemical production [1]. Corn stover, a lignocellulosic feedstock, is one of the most important agricultural residues available in high quantities with about 900 million tons produced in 2018 in China according to Ministry of Agriculture and Rural Affairs. Many studies have examined corn stover applied in different fields, such as generating electricity [2], biofuel [3] and biochemical production [4], and biological feed [5]. Especially, lactic acid (LA) is an important commodity chemical and also a monomer compound to produce biodegradable and biocompatible polylactic acid (PLA), which provides a sustainable alternative to petroleum-derived products [6]. The corn stover usually contains 37.5% cellulose, 22.4% hemicellulose, and 17.6% lignin, which can be hydrolyzed into hexose and pentose for fermentation [7].

However, lignocellulose hydrolysis and utilization remain considerable challenges in lignocellulose-derived biofuel and biochemical production [8]. At present, the main obstacles include lignocellulose pretreatment and hydrolysis, as well as the efficient fermentation of pentose derived from lignocellulosic hydrolysates into LA. Many studies have evaluated lignocellulose degradation and biofuel and biochemical production by using physical [9,10], chemical, and biological [8] pre-treatments. Chemical methods are the most common pretreatment methods, including acid treatment, alkaline treatment, alkaline/oxidative treatment, wet oxidation, and ozonolysis [11,12,13]. Xylose limits the conversion from the hydrolysate of lignocellulose into LA [14]. Therefore, it is necessary to isolate or construct the strains that can efficiently utilize pentose. Several lactic acid bacteria (LAB) such as *Lactobacillus pentosus* [4], *Lactobacillus brevis* [15], *Enterococcus mundtii* QU 25 [16], and *Enterococcus faceium* QU 50 [17] have been reported to ferment xylose via the phosphoketolase pathway (hetero-fermentation pathway). Recently, including *Lactobacillus* strains [4,15,18], *Bacillus coagulans* [12,19,20,21], and *Pediococcus acidilactici* [22,23], they have also been reported to produce high-titer LA from lignocellulosic materials. These strains with robust inhibition tolerance were found to be suitable for lignocellulose-derived LA production and were engineered for chemical production because of their thermophilic growth characteristics (except *Lactobacillus* strains) and strong pentose homofermentative activity.

Acid-pretreatment methods were used to efficiently produce LA. LA yield and titer produced from oil palm empty fruit bunch (OPEFB) acid hydrolysate reached 0.97 g/g and 59.2 g/L through *B. coagulans* fermentation, respectively [24]. LA yield and titer obtained from acid-pretreated wheat stover reached 0.46 g/g (wheat stover) and 38.73 g/L, respectively, via *B. coagulans* IPE22 fermentation [20]. Excellent LA production was obtained from sulfuric acid-pretreated and biodetoxified corn stover through *Pediococcus acidilactici* DQ2 fermentation [25] with a good LA titer of 101.9 g/L and poor yield of a mere 0.38 g/g stover as *P. acidilactici* DQ2 cannot utilize xylose. A high titer (104.4 g/L) of L-LA was obtained from dilute acid-pretreated and biodetoxified corn stover through an engineered *P. acidilactici* TY112 (CGMCC 8664) strain fermentation, and the yield of L-LA reached 0.72 g/g glucose from total corn stover regardless of xylose unavailability [26]. However, the acid-pretreated method can lead to hemicellulose loss and cannot efficiently remove lignin [27,28]. Based on this, alkali-pretreated methods were exploited and applied. In our previous studies, *B. coagulans* LA204 and *L. pentosus* FL0421 could efficiently utilize corn stover and corncob to produce LA [4,12,13]. By using NaOH-pretreated and washed corn stover, the LA yield and titer increased to 0.68 g/g substrate and 97.6 g/L, respectively, through *B. coagulans* LA204 fed-batch fermentation [12]. The LA yield and titer obtained from alkali-pretreated corn stover reached 0.66 g/g corn stover and 92.3 g/L, respectively, via *L. pentosus* FL0421 simultaneous saccharification and fermentation (SSF) [4]. By using *B. coagulans* LA204 fed-batch fermentation, the lactic acid titer and yield produced from 16% (*w*/*w*) NaOH-pretreated corncob were 122.99 g/L and 0.77 g/g, respectively, and those produced from 16% NH_3_-H_2_O_2_-pretreated and washed corncob were 118.60 g/L and 0.74 g/g corncob, respectively [13].

*Pediococcus acidilactici* PA204 with high temperature tolerance (32–47 °C) and efficient xylose conversion into lactic acid was used for high titer lactic acid production at the high solids loading of corn stover through the simultaneous saccharification and fermentation (SSF). As probiotics, *P*. *acidilactici* PA204 produces bacteriocin, which has a good inhibitory effect on some pathogenic microorganisms in the intestinal tract [29]. Additionally, this strain has long been announced to be safe as a probiotic strain that can be used in food and drugs by the food and drug administration (FDA) [30].

In this study, we developed a fed-batch SSF process for LA production using corn stover as a carbon source through *P. acidilactici* PA204 fermentation, and found that this strain produced LA from 12% (*w*/*w* loading) NaOH-pretreated and washed corn stover with a titer of 92.01 g/L and yield of 0.77 g/g stover. It also produced acetic acid from the same corn stover with a titer of 10.03 g/L and yield of 0.08 g/g stover under nonsterile conditions. Our results provide a practical process for lactic acid production from lignocellulose residues. This study reveals that *P. acidilactici* PA204 is a promising candidate strain for high-yield and -titer lignocellulose-derived LA production.

## 2. Materials and Methods

### 2.1. Raw Materials and Enzyme

Corn stover was grown in the Northeast of China and harvested in the autumn of 2016. After harvesting, corn stover was cleaned, dried, and sieved with the 80-mesh and pretreated at 75 °C for 3 h with 5% sodium hydroxide (NaOH) at a 20% (*w*/*w*) loading. The resultant slurry was washed with water until the pH decreased to 8.0, and then filtered to a moisture content of 20% (*w*/*w*). The raw corn stover consisted of 37.12 ± 0.32 cellulose, 29.60 ± 0.26 hemicellulose, and 20.80 ± 0.56 lignin. The pretreated corn stover contained 51.34 ± 0.57 cellulose, 27.20 ± 0.34 hemicellulose, and 8.31 ± 0.89 lignin. The pretreated and washed corn stover contained 60.01 ± 0.51 cellulose, 27.33 ± 0.21 hemicellulose, and 6.87 ± 0.66 lignin. The cellulase used in this study was Cellic CTec2 (Novozymes, Denmark) containing cellulase, β-glucosidase, and xylanase, with its cellulose activity of 250 filter paper units (FPU)/mL.

### 2.2. Medium and Strain

The De Man-Rogosa-Sharpe (MRS) medium was used for seed culturing. MRS medium contained 10 g of peptone, 10 g of beef extract, 5 g of yeast extract, 20 g of dextrose, 5 g of sodium acetate trihydrate, 1 g of polysorbate 80, 2 g of dipotassium phosphate, 2 g of triammonium citrate, 0.25 g of magnesium sulfate heptahydrate, and 0.05 g of manganese sulfate tetrahydrate in 1 L deionized water [31]. The medium and water were autoclaved at 115 °C for 20 min. *Pediococcus acidilactici* PA204 was isolated and stored in our laboratory. Activation cultures were carried out in MRS medium at 37 °C for 24 h.

### 2.3. Simultaneous Saccharification and Fermentation (SSF), and NaOH-Pretreated Corn Stover

The mixture of 4% NaOH-pretreated and washed corn stover, 20 g/L corn steep powder, and cellulase at a concentration of 30 FPU/g stover was inoculated with 200 mL seed culture to establish the SSF process in a 2 L volume. Fermentation was carried out at 37 °C for 60 h with agitation at 150 rpm. The pH was maintained at 6.0 by automatic feeding of 10 M NaOH solution.

Fed-batch fermentations were performed with 8% pretreated and washed corn stover, a cellulase concentration of 30 FPU/g stover, 20 g/L corn steep powder, and 200 mL seed culture (*v/v*) in a total volume of 2 L. During fermentation, the washed corn stover was continuously fed at 24 h, and the final concentration of pretreated and washed corn stover reached 12% (*w/w*) or 15% (*w/w*) and total fermentation volume reached approximately 3 L. Enzyme feeding for all fermentations is illustrated in Figure 3 with the final enzyme concentration of 30 FPU/g stover. Samples were collected every 6 or 12 h. The concentrations of LA, acetic acid, glucose, and xylose were determined by HPLC. The fed corn stover was not sterilized.

### 2.4. Analysis of Sugars, Acids, and Biomass

Glucose, xylose, LA, acetic acid, and formic acid were analyzed using an Agilent 1200 HPLC system equipped with an RID-10A detector or an SPD-20A detector, and a Bio-Rad Aminex HPX-87H column with a column temperature of 40 °C. The mobile phase was 5 mM H_2_SO_4_, and the flow rate was set as 0.6 mL/min. The LA yield was defined as the produced LA (g) per total sugar or total corn stover (g). All samples were centrifuged and filtered through a 0.22 μm membrane prior to loading. The strain biomass was counted by the dilution plate method.

## 3. Results and Discussion

### 3.1. Effects of Temperature, pH, Carbon Source, and Nitrogen Source on LA Production

**Figure 1:**
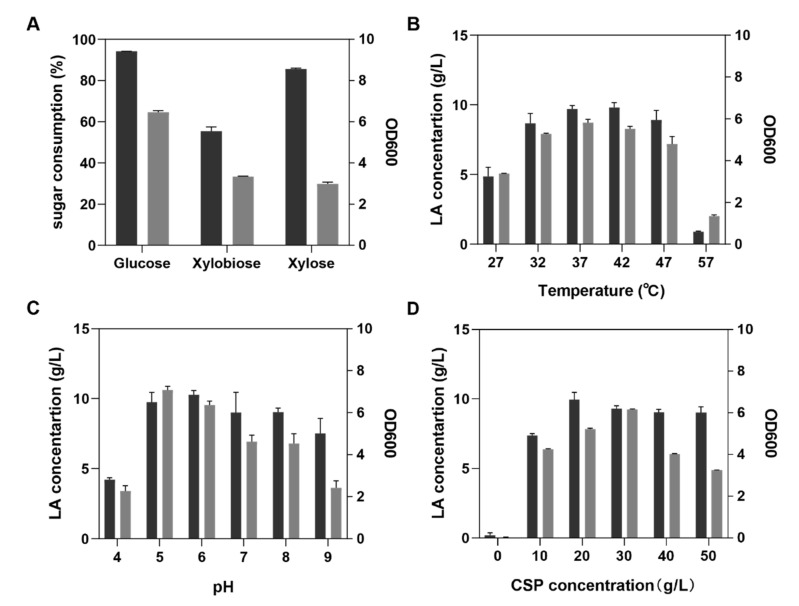
General features of *P. acidilactici* PA204 fermentation. (**A**) Lactic Acid (LA) production efficiency from glucose, xylose, and xylobiose media. Culture contains 10 g/L of each sugar and 5 g/L of yeast extract, pH 6.0; fermentation was carried out at 37 °C. (**B**) Effect of temperature on LA fermentation. Culture contains 10 g/L glucose and 5 g/L yeast extract (pH 6.0). (**C**) Effect of pH on LA fermentation. Culture contains 10 g/L glucose and 5 g/L yeast extract; fermentation was carried out at 37 °C. (**D**) Effect of CPS at different concentrations on LA production. CSP: Corn steep powder. Culture contains 10 g/L glucose (pH 6.0). Fermentation was carried out at 37 °C. Black bars: Sugar consumption efficiency and LA concentration; gray bars: OD600.

*P. acidilactici* PA204, which can efficiently utilize glucose to produce lactic acid, was isolated and stored in the Fermentation Engineering Laboratory at Huazhong Agriculture University. In this study, the fermentation abilities of *P. acidilactici* PA204 to glucose, xylose, and xylobiose were tested in flasks. The sugar consumptions of glucose, xylose, and xylobiose media reached 94%, 86%, and 55% in 10 g/L sugar concentration, respectively. We found that the cell count in the glucose media was higher than those of other sugars (Figure 1A). These results suggested that *P. acidilactici* PA204 might have a strong ability to ferment lignocellulosic hydrolysates (glucose and xylose) into LA. We further examined the effect of temperature on *P. acidilactici* PA204 LA fermentation in glucose media. As shown in Figure 1B, the maximum LA yields (0.97 and 0.98 g/g sugar) were achieved at 37 °C and 42 °C, respectively, while lower yields were found at other temperatures. The value of OD600 reached the highest (5.82 and 5.52) at 37 °C and 42 °C, respectively. However, at 57 °C, the value of OD600 was only 1.34; thus, lactic acid can barely be produced. The effect of initial pH values on LA fermentation was also examined. The results indicated that an initial pH of 6.0 was optimal for lactic acid fermentation by this strain. As can be seen from Figure 1C, a higher LA titer was obtained at pH 6.0 (9.74 g/L vs. 10.27 g/L, pH 5.0 vs. 6.0). In addition, the effect of nitrogen source concentration on LA fermentation was examined with 10 g/L glucose as the carbon source and different concentrations of corn steep powder as the nitrogen source (Figure 1D). The results indicated that the highest LA concentration (9.92 g/L) was produced from corn steep powder (at the concentration of 20 g/L) and that the cell count in the media containing 20 and 30 g/L concentrations of corn steep powder was higher than that in other media, suggesting that the effects of nitrogen sources concentrations on lactic acid production efficiency coincided with those on cell growth efficiency. Moreover, the increase in nitrogen source concentration did not significantly affect the production of LA. These results indicated that corn steep powder can be used by *P. acidilactici* PA204 as an inexpensive nitrogen source for LA production and that the concentration of 20 g/L can be determined as the optimal nitrogen source concentrations.

### 3.2. High-Titer and High-Yield LA Production from NaOH-Pretreated Corn Stover through SSF

NaOH pretreatment was reported to be an efficient method to remove lignin and destroy the structure of lignocellulose in our previous studies [12]. In this study, we selected NaOH pretreatment to remove lignin from corn stover and determined the compositional changes of corn stover before and after NaOH pretreatments. The higher the solubilization and reduction of lignin, the greater the cellulose composition increase of pre-treated corn stover [28]. As shown in Table 1, the solid fraction from NaOH pre-treated and washed corn stover showed a significant 61.66% increase in cellulose composition due to the sharp 66.97% decline in the percentage of lignin, and a lower marked 7.67% decrease in hemicellulose composition. The results indicated that NaOH pretreatment is an efficient method to remove lignin and retain cellulose and hemicellulose.

**Figure 2:**
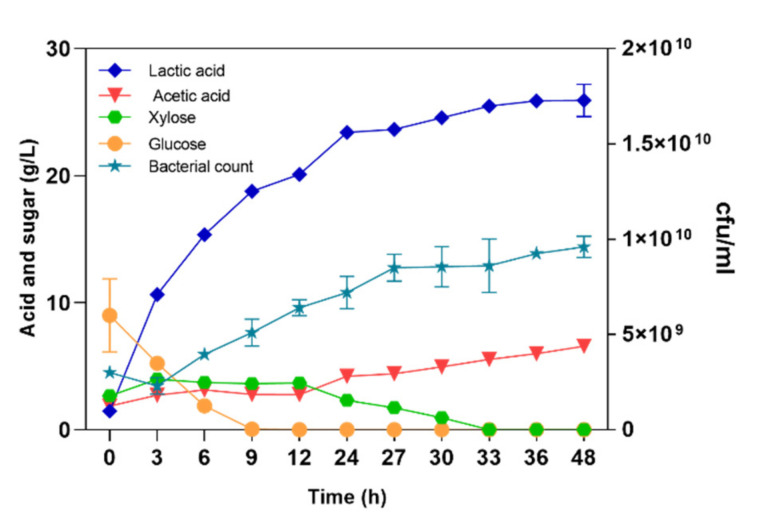
Lactic acid production from NaOH-pretreated and washed corn stover (4% *w*/*w*). The final cellulase concentration was 30 FPU/g stover. Diamonds indicate lactic acid. Inverted triangles represent acetic acid. Dots indicate glucose. Polygons represent xylose. Pentacle represent bacterial count. The three fermentation stages are presented in the figures.

In the initial SSF experiment, 4% (*w*/*w*) NaOH-pretreated and washed corn stover and 20 g/L corn steep powder were the carbon source and nitrogen source, respectively. Cellulase was added at the beginning of fermentation, and the final enzyme concentration used was 30 FPU/g stover. The 10 M NaOH solution was used as the neutralizer. Initially, LA was produced rapidly. At hour 6 during fermentation, the LA titer reached 15.35 g/L, and the productivity reached 2.59 g/L/h (Figure 2), suggesting that for the first 6 h, corn stover rapidly degraded by cellulase and xylanase into glucose and xylose. Then, *P. acidilactici* PA204 quickly utilized sugar to produce LA. However, at hour 48 during fermentation, the LA titer reached 25.92 g/L, the yield reached 0.65 g/g stover, and the average productivity was 0.54 g/L/h (Table 2). The average productivity at hour 48 was significantly lower than that at the early fermentation stage. At hour 33 during fermentation, glucose and xylose were hardly detected and no significant increase in LA titer was observed from hour 33 (25.48 g/L) to hour 48 (25.92 g/L). During fermentation, the amounts of bacteria was gradually increased, and reached 9.1 × 10^9^ cfu/mL at hour 27. After that, the increase in bacterium count tended to be stable, indicating the end of fermentation.

### 3.3. High-Titer and High-Yield LA Production through Fed-Batch Fermentation

**Figure 3:**
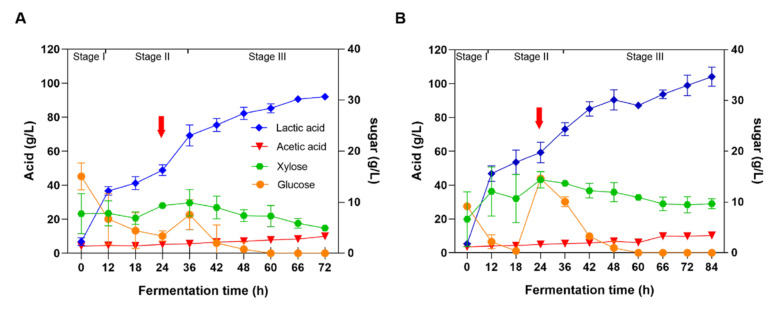
Lactic acid production from NaOH-pretreated and washed corn stover. (**A**) Initial 8% (*w*/*w*) NaOH-pretreated and washed corn stover was fed with the stover treated in the same manner at hour 24 to reach the final concentration of 12.0% (*w*/*w*). (**B**) Initial 8% (*w*/*w*) NaOH-pretreated and washed corn stover was fed with the stover treated in the same manner at hour 24 to reach the final concentration of 15.0% (*w*/*w*). Substrate and enzyme feedings are indicated by red arrows. The final cellulase concentration in both experiments was 30 FPU/g stover. Diamonds indicate lactic acid. Inverted triangles represent acetic acid. Dots indicate glucose. Polygons represent xylose. The three fermentation stages are presented in the figures.

A good LA yield was obtained from 4% (*w*/*w*) NaOH-pretreated and washed corn stover, but the LA titer was not sufficient. Thus, a high solid loading of lignocellulosic materials was necessary to increase the LA titer. Considering the substance inhibition effect, the fed-batch experiment was carried out to increase the LA titer and yield. In two fed-batch experiments, 8% (*w*/*w*) NaOH-pretreated and washed corn stover (total of 2 L) was inoculated with 10% seed culture and supplemented with 30 FPU/g of the cellulase solution. At hour 24 during fermentation, the pretreated and washed corn stover was continuously fed to reach the final corn stover concentration of 12% (*w*/*w*) or 15% (*w*/*w*), and the enzyme was fed to reach the final enzyme concentration of 30 FPU/g stover (Figure 3). Finally, the titers of LA produced from high solid loadings of NaOH-pretreated and washed corn stover were tested. As Figure 3A shows, the LA yield from 12% solid loading reached (*w*/*w*) 0.77 g/g stover, LA titer reached 92.01 g/L, and the average productivity was 1.28 g/L/h. The acetic acid titer reached 10.03 g/L and its yield was 0.08 g/g stover (Table 2). However, the LA yield from 15% solid loading (*w*/*w*) reached 0.69 g/g stover, the titer reached 104.11 g/L, and the average productivity was 1.24 g/L/h (Figure 3B). The acetic acid titer reached 10.28 g/L and its yield was 0.07 g/g stover (Table 2).

Three basic stages were identified in the LA fed-batch fermentation process using corn stover as the substrate (Figure 3). During the first stage (0–12 h), LA was quickly produced in all two experiments, and during this period, the productivity was 3.05 g/L/h (12% *w*/*w* loading, Figure 3A), and 3.90 g/L/h (15% *w*/*w* loading, Figure 3B), respectively. These results indicated that LA productivity was dependent on the concentration of corn stover (8% *w*/*w* loading) at this stage. Therefore, both glucose and xylose were rapidly released, and then consumed (Figure 3). During the second stage (12–36 h) at 12% *w*/*w* corn stover loading (Figure 3A), LA production slowed down before being fed with corn stover (1.01 g/L/h, 12–24 h). However, after being fed with stover and enzyme, LA significantly increased (1.69 g/L/h, 24–36 h). Similarly, during the second stage (12–36 h) at 15% *w*/*w* corn stover loading (Figure 3B), LA production slowed down before corn stover feeding (1.04 g/L/h, 12–24 h) and LA increased (1.15 g/L/h, 24–36 h) after stover and enzymes feeding. Interestingly, LA productivity at 12% *w*/*w* stover feeding was slightly higher than that at 15% *w*/*w* stover feeding (1.69 g/L/h vs. 1.15 g/L/h, Figure 3), indicating that LA productivity was dependent on the concentration of corn stover, and that the inhibitors in the corn stover might inhibit LA production [4]. The sugars consumption was observed. Glucose was found to be quickly released and consumed completely. However, xylose was accumulated and was not utilized fully at the end of fermentation (Figure 3). The result may be produced due to substrate inhibition. When a large amount of xylose was continuously released, it could not be consumed and utilized by *P. acidilactici* PA204 in time. Phenolic compounds in corn stover might inhibit utilization of xylose by *P. acidilactici* PA204. The underlying mechanism needed to be further investigated.

During the third stage (36 h to the end of fermentation), LA titer was increased continuously and slowly in fed-batch experiments, and significant differences in LA titer and productivity were observed between the 12% *w*/*w* feeding group (92.01 g/L and 0.634 g/L/h) and 15% *w*/*w* feeding group (104.11 g/L and 0.646 g/L/h). The glucose was consumed completely at hour 36 and xylose remained at a certain concentration until the end of fermentation (5.29 g/L) in the 12% *w*/*w* feeding group (Figure 3A). However, in the 15% *w*/*w* feeding group, the glucose was consumed completely at hour 60 and xylose remained at the concentration of 9.66 g/L (Figure 3B). Although the concentration of lactic acid was increased, lactic acid yield was slightly decreased. These results suggested that a high solid loading of corn stover could release more sugars and increase the yield of lactic acid to some extent. However, a high solid loading caused the delayed consumption of glucose and retention of more xylose. The possible reason might lie in that more pretreated corn stover might result in more inhibitors, as well as a substrate effect.

As shown in Table 2, the LA titer, yield, and productivity at 12% (*w*/*w*) NaOH-pretreated and washed corn stover feeding were 92.01 g/L, 0.77 g/g stover, and 1.28 g/L/h, respectively, and at 15% (*w*/*w*) NaOH-pretreated and washed stover feeding, they were 104.11 g/L, 0.69 g/g stover, and 1.24 g/L/h, respectively. Generally, the chemical pretreatment method could induce inhibitors generation, whereas the washing method could obviously reduce the inhibitor concentration and improve LA production. However, the major disadvantage of the washing method is the substantial volume of waste washing water generated by inhibitor removal. In the industrial production process, wastewater should be strictly limited because of the high cost of wastewater treatment. Therefore, other methods including biological detoxification, pretreatment with few inhibitors, and screening of inhibitor-tolerant strains need to be developed in order to increase the LA titer and yield by using unwashed pretreated corn stover.

## 4. Conclusions

In this study, we produced high-titer and high-yield LA from NaOH-pretreated and washed corn stover through *P. acidilactici* PA204 fermentation. Importantly, *P. acidilactici* PA204 was found to specifically ferment xylose to produce lactic acid. An efficient SSF fed-batch process for LA production from NaOH-pretreated and washed corn stover by *P. acidilactici* PA204 has been established at 37 °C and pH 6.0 with a cellulase activity of 30 FPU/g stover and 20 g/L corn steep powder in a 5 L bioreactor. The lactic acid titer, yield, and productivity were 92.01 g/L, 0.77 g/g stover, and 1.28 g/L/h at 12% (*w*/*w*) NaOH-pretreated and washed stover feeding, respectively, and they were 104.11 g/L, 0.69 g/g corncob, and 1.24 g/L/h at 15% NaOH-pretreated and washed stover feeding, respectively. What is more, fermentation without sterilization has advantages in reducing production cost and energy consumption. This study develops a feasible fed-batch SSF process for LA production from corn stover and provides a promising candidate strain for high-titer and -yield lignocellulose-derived LA production.

## Figures and Tables

**Table 1:** The composition of different corn stover in this study. A: Raw corn stover. B: NaOH pre-treated corn stover. C: NaOH pre-treated corn stover and washed corn stover.

Corn Stover	Cellulose (%)	Hemicellulose (%)	Lignin (%)
A	37.12 ± 0.32	29.60 ± 0.26	20.80 ± 0.56
B	51.34 ± 0.57	27.20 ± 0.34	8.31 ± 0.89
C	60.01 ± 0.51	27.33 ± 0.21	6.87 ± 0.66

**Table 2:** Summary of lactic acid fermentation by *P. acidilactici* PA204 using NaOH-pretreated and washed corn stover with washing as the carbon source. 4% stover: Simultaneous saccharification and fermentation (SSF) batch experiment. 12% and 15% stover: Fed-batch experiments.

Experiments	4% Stover	12% Stover	15% Stover
Lactic acid titer (g/L)	25.92	92.01	104.11
Lactic acid yield (g/g stover)	0.65	0.77	0.69
Lactic acid productivity (g/L/h)	0.54	1.28	1.24
Acetic acid titer (g/L)	6.57	10.03	10.28
Acetic acid yield (g/g stover)	0.16	0.08	0.07
Acetic acid productivity (g/L/h)	0.14	0.14	0.12
